# Low preoperative regional cerebral oxygen saturation in hemodialysis patients

**DOI:** 10.1186/s40981-017-0084-7

**Published:** 2017-04-08

**Authors:** Shino Matsukawa, Miho Hamada, Toshiyuki Mizota

**Affiliations:** Department of Anesthesia, Kyoto University Hospital, 54 Shogoin-Kawahara-Cho, Sakyo-Ku, Kyoto, 606-8507 Japan

**Keywords:** Hemodialysis, Regional cerebral oxygen saturation, Cardiac surgery

## Abstract

**Background:**

Regional cerebral oxygen saturation (rSO_2_) monitoring by near-infrared spectroscopy provides valuable information regarding cerebral oxygen delivery, and it has been increasingly used in cardiovascular surgery. Although it has been shown that dialysis-dependent patients [hemodialysis (HD) patients] suffer from low cerebral perfusion, limited information is available on cerebral tissue oxygenation levels in HD patients.

**Findings:**

In this retrospective study, the preoperative rSO_2_ values in 9 HD patients undergoing coronary artery bypass graft surgery were compared with those in 40 non-HD patients. HD patients had lower preoperative rSO_2_ values than non-HD patients (median: 46 vs. 68%, respectively, *P* < 0.001). Despite adjusting for age, hemoglobin concentration, and left ventricular ejection fraction using multivariable linear regression, HD showed a strong association with low rSO_2_ (estimated coefficient: −20.4, *P* < 0.001).

**Conclusions:**

HD showed a strong association with low preoperative rSO_2_ values in patients undergoing coronary artery bypass graft surgery, even after adjusting for known factors that affect rSO_2_ values, including age, hemoglobin concentration, and cardiac systolic function. Further research is required to elucidate the mechanisms decreasing rSO_2_ values in HD patients.

## Findings

### Introduction

Regional cerebral oxygen saturation (rSO_2_) monitoring by near-infrared spectroscopy has been increasingly used to assess the adequacy of cerebral oxygen delivery in patients undergoing cardiovascular surgery [1, 2]. Previous studies have shown that rSO_2_, measured by near-infrared spectroscopy, is affected by several factors, including age, hemoglobin concentration, and cardiac function [3–7].

It has been shown that end-stage renal disease patients on hemodialysis (HD) suffer from low cerebral perfusion, which may contribute to cognitive deficits and high stroke prevalence observed in dialysis-dependent patients (HD patients) [8]. However, limited information is currently available on cerebral tissue oxygenation levels in HD patients.

Here, we hypothesized that the preoperative rSO_2_ values in HD patients undergoing cardiac surgery is lower than those in non-HD patients, reflecting low cerebral blood flow. The current study was designed to compare the preoperative rSO_2_ values in HD patients and non-HD patients undergoing coronary artery bypass grafting (CABG).

## Methods

Our study was approved by the ethics committee of Kyoto University (approval number: R0719), and the requirement for written informed consent was waived. We included adult (≥20 years of age) patients who underwent isolated CABG at Kyoto University Hospital between January 1, 2012 and December 31, 2015. Patients who concurrently underwent cardiac valvular or aortic surgery were excluded. Patients without available rSO_2_ values taken before administration of oxygen or anesthetic drugs were also excluded.

Specific data from the electronic medical records of participants, including age, gender, body mass index, comorbidities, and preoperative examination findings, were retrospectively collected. Left ventricular ejection fraction (LVEF) was derived from preoperative transthoracic echocardiography records. For hemoglobin concentration values, the most recent value measured before the surgery was used.

Patients received no premedication. Upon entering the operating room, preoperative rSO_2_ and arterial oxygen saturation by pulse oximetry (SpO_2_) were obtained before administration of oxygen or anesthetic drugs. rSO_2_ values were detected using the INVOS 5100 system (Somanetics, Troy, MI) with probes containing light sources, each providing two continuous wavelengths of near-infrared light (730 and 810 nm) that reach a brain area corresponding to the junction between the anterior and middle cerebral artery vascularization territory. Two probes were attached to the right and the left sides of the forehead; the preoperative rSO_2_ values were defined as the mean of rSO_2_ values obtained from these two probes.

Data were analyzed using the statistical program R (http://cran.r-project.org). Continuous data are presented as median (interquartile range), and categorical variables are expressed as a number (percentage). Differences between groups were compared using the Mann–Whitney *U* test for continuous variables. For categorical variables, Pearson’s chi-square or Fisher’s exact tests were used as appropriate. Because it has been shown that rSO_2_ is affected by age, hemoglobin concentration, and cardiac function [3–7], we used multivariable linear regression analysis to assess the independent impact of HD on rSO_2_ values after adjustment for these factors. We constructed a linear regression model with rSO_2_ as a dependent variable and HD status, age, hemoglobin concentration, and LVEF as independent variables, and beta coefficients were calculated for all independent variables. All statistical tests were two-tailed, and the statistical significance was set at a *P* value of <0.05.

## Results

A total of 80 patients underwent isolated CABG during the study period. Of these, 31 patients were excluded because of the lack of rSO_2_ measurements before administration of oxygen or anesthetic drugs; the remaining 49 patients were included in the analysis. Among these 49 patients, 9 patients (18.4%) received HD preoperatively. Duration of HD in HD patients ranged from 2 to 14 years; 4 patients (44.4%) underwent HD for ≥10 years. Patient characteristics stratified by HD status are presented in Table 1. HD patients had significantly higher prevalence of diabetes mellitus and arteriosclerosis obliterans and significantly lower hemoglobin concentrations than non-HD patients. Although it did not reach statistical significance, LVEF in HD patients tended to be lower than that in non-HD patients.

**Table 1:** Patient characteristics stratified by HD

	Non-HD patients (*n* = 40)	HD patients (*n* = 9)	*P* value
Age (years)	73 (64–78)	73 (69–77)	0.846
Female gender	7 (17.5%)	2 (22.2%)	0.741
Body mass index (kg/m^2^)	23.4 (21.7–25.3)	22.2 (19.4–24.2)	0.245
Hypertension	30 (75.0%)	7 (77.8%)	0.861
Diabetes mellitus	13 (32.5%)	9 (100.0%)	<0.001
ASO	1 (2.5%)	4 (44.4%)	<0.001
Hemoglobin concentration (g/dL)	12.7 (11.9–14.1)	10.1 (9.7–11.8)	0.001
LVEF (%)^a^	66.0 (55.3–72.0)	50.0 (29.9–73.9)	0.398

*HD* hemodialysis, *ASO* arteriosclerosis obliterans, *LVEF* left ventricular ejection fraction, *rSO*_*2*_ regional cerebral oxygen saturation

^a^LVEF was missing in one non-HD patient

**Fig. 1:**
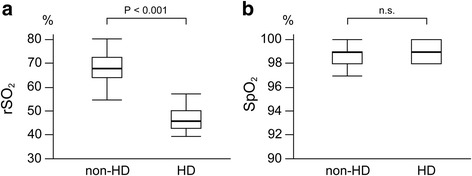
Box plots of preoperative rSO_2_ (**a**) and SpO_2_ (**b**) values stratified by HD. *Horizontal bold lines* represent median values. *rSO*_*2*_ regional cerebral oxygen saturation, *SpO*_*2*_ arterial oxygen saturation of pulse oximetry, *HD* hemodialysis, *n.s.* not significant

Distributions of preoperative rSO_2_ and SpO_2_ values stratified by HD status are shown in Fig. 1. HD patients had significantly lower rSO_2_ values than non-HD patients [46% (43–50%) vs. 68% (64–73%), *P* < 0.001; Fig. 1a]. No patients had an rSO_2_ value of ≤50% in the non-HD group, whereas 7 out of 9 HD patients (77.8%) had an rSO_2_ value of ≤50%. SpO_2_ values of study participants ranged from 94–100% and were comparable between groups (*P* = 0.625; Fig. 1b).

Multivariable linear regression analysis was performed to assess the impact of HD on preoperative rSO_2_ values after adjustment for age, hemoglobin concentration, and LVEF, which have been shown to affect rSO_2_ in previous studies [3–7]. HD maintained a strong association with low rSO_2_, even after adjusting for age, hemoglobin concentration, and LVEF. The estimated coefficient for HD in the multivariable model was −20.4 (95% confidence interval: −26.3 to −14.4) (Table 2).

**Table 2:** Multivariable linear regression analysis for the association of HD and rSO_2_, adjusting for age, hemoglobin concentration, and LVEF

	Beta (95% confidence interval)	*P* value
Intercept	60.9 (39.0 to 82.8)	
HD	−20.4 (−26.3 to −14.4)	<0.001
age	−0.03 (−0.22 to 0.16)	0.753
Hemoglobin concentration	0.38 (−0.78 to 1.55)	0.510
LVEF^a^	0.08 (−0.05 to 0.20)	0.236

*HD* hemodialysis, *rSO*_*2*_ regional cerebral oxygen saturation, *LVEF* left ventricular ejection fraction

^a^One non-HD patient with missing LVEF data was excluded from this analysis

## Discussion

This study demonstrated that HD patients had significantly lower preoperative rSO_2_ values, even after adjusting for known factors that affect rSO_2_, including age, hemoglobin concentration, and LVEF.

Several reports have suggested that patients undergoing HD have low rSO_2_ values, reflecting low cerebral perfusion [9, 10]; however, they did not assess cardiac function, which is known to affect rSO_2_ values [7]. In our study, we were able to compare rSO_2_ values in HD and non-HD patients after adjusting for LVEF because most patients underwent preoperative transthoracic echocardiography. Although HD patients had significantly lower hemoglobin concentrations than non-HD patients, and LVEF in HD patients tended to be lower, the estimated coefficient for HD in the multivariable linear regression analysis, adjusting for age, hemoglobin concentration, and LVEF, was −20.4. This means that HD patients had 20.4% lower rSO_2_ values than non-HD patients after adjusting for these factors, indicating that low rSO_2_ values in HD patients are, at least in part, because of factors other than age, anemia, or cardiac systolic function. Although it is not possible to determine the specific cause of low rSO_2_ values in HD patients in our study, possible explanations are as follows: (1) metabolic acidosis frequently seen in HD patients decreases affinity between hemoglobin and oxygen [11] and decreases microcirculatory oxygen saturation. (2) The acute intravascular volume loss and fluid shifts that occur during dialysis induce cerebral edema and decrease intracerebral blood pressure, blood velocity, and cerebral perfusion [12]. (3) Cerebral atrophy seen in HD patients [13] might increase the thickness of the cerebrospinal fluid layer, which decreases the intensity of near-infrared light that the detector can receive, thereby, decreasing rSO_2_ values [5]. Further research is required to elucidate the mechanisms by which the rSO_2_ values are decreased in HD patients.

It is important to determine adequate target values of rSO_2_ to guide intraoperative management in HD patients. rSO_2_ monitoring may be used to guide hemodynamics and CPB management during cardiac surgery by adjusting therapy based on relative changes from the preoperative baseline (i.e., to maintain relative rSO_2_ > 80% of baseline) [14]. However, this concept might allow too low rSO_2_ values and be harmful for majority of HD patients, who frequently have abnormally low baseline rSO_2_ values. Future research should be conducted to establish the appropriate target value of rSO_2_ in HD patients.

This study had certain limitations, primarily based on its retrospective design. We used only preoperative LVEF to evaluate cardiac function because data on diastolic function or cardiac output were not available in most patients. In addition, we could not adjust for the influence of some factors, which are suggested to be related to rSO_2_, including partial pressure of carbon dioxide in arterial blood, central venous pressure, skull thickness, and area of cerebrospinal fluid layer [4, 5]. The number of patients was small; however, it was still sufficient to support our hypothesis.

## Conclusions

In conclusion, this study demonstrated that HD showed strong associations with low preoperative rSO_2_ values in patients undergoing CABG, even after adjusting for known factors that affect rSO_2_ values, including age, hemoglobin concentration, and cardiac systolic function.

## Abbreviations

CABG: Coronary artery bypass grafting
HD: Hemodialysis
LVEF: Left ventricular ejection fraction
rSO_2_: Regional cerebral oxygen saturation
SpO_2_: Arterial oxygen saturation by pulse oximetry
